# The Music-In-Noise Task (MINT): A Tool for Dissecting Complex Auditory Perception

**DOI:** 10.3389/fnins.2019.00199

**Published:** 2019-03-14

**Authors:** Emily B. J. Coffey, Isabelle Arseneau-Bruneau, Xiaochen Zhang, Robert J. Zatorre

**Affiliations:** ^1^Department of Psychology, Concordia University, Montreal, QC, Canada; ^2^Laboratory for Brain, Music and Sound Research (BRAMS), Montreal, QC, Canada; ^3^Centre for Research on Brain, Language and Music (CRBLM), Montreal, QC, Canada; ^4^Centre for Interdisciplinary Research in Music Media and Technology (CIRMMT), Montreal, QC, Canada; ^5^Montreal Neurological Institute, McGill University, Montreal, QC, Canada; ^6^Department of Biomedical Engineering, School of Medicine, Tsinghua University, Beijing, China

**Keywords:** hearing-in-noise, auditory stream segregation, musical training, multilingualism, neuroplasticity, interindividual variability, auditory working memory, skill assessment tool

## Abstract

The ability to segregate target sounds in noisy backgrounds is relevant both to neuroscience and to clinical applications. Recent research suggests that hearing-in-noise (HIN) problems are solved using combinations of sub-skills that are applied according to task demand and information availability. While evidence is accumulating for a musician advantage in HIN, the exact nature of the reported training effect is not fully understood. Existing HIN tests focus on tasks requiring understanding of speech in the presence of competing sound. Because visual, spatial and predictive cues are not systematically considered in these tasks, few tools exist to investigate the most relevant components of cognitive processes involved in stream segregation. We present the Music-In-Noise Task (MINT) as a flexible tool to expand HIN measures beyond speech perception, and for addressing research questions pertaining to the relative contributions of HIN sub-skills, inter-individual differences in their use, and their neural correlates. The MINT uses a match-mismatch trial design: in four conditions (Baseline, Rhythm, Spatial, and Visual) subjects first hear a short instrumental musical excerpt embedded in an informational masker of “multi-music” noise, followed by either a matching or scrambled repetition of the target musical excerpt presented in silence; the four conditions differ according to the presence or absence of additional cues. In a fifth condition (Prediction), subjects hear the excerpt in silence as a target first, which helps to anticipate incoming information when the target is embedded in masking sound. Data from samples of young adults show that the MINT has good reliability and internal consistency, and demonstrate selective benefits of musicianship in the Prediction, Rhythm, and Visual subtasks. We also report a performance benefit of multilingualism that is separable from that of musicianship. Average MINT scores were correlated with scores on a sentence-in-noise perception task, but only accounted for a relatively small percentage of the variance, indicating that the MINT is sensitive to additional factors and can provide a complement and extension of speech-based tests for studying stream segregation. A customizable version of the MINT is made available for use and extension by the scientific community.

## Introduction

A person listening to music over earbuds on a busy city street is a common sight in today’s world. But the apparent ease of the listener’s ability to enjoy the music despite the many irrelevant surrounding noises belies the complexity of the auditory cognitive mechanisms at play. The auditory system’s ability to separate the important signals from the background has been termed stream segregation, or more broadly, Auditory Scene Analysis (Bregman, 1990, 2015; Shamma and Micheyl, 2010) and has a long history in experimental psychology, audiology, and speech sciences. It represents a fundamental and very complex computational problem, because the input to the system—an undifferentiated mix of all the various sound waves impinging on the eardrum—is inherently ambiguous (Pressnitzer et al., 2011), and thus requires significant resources, including prior knowledge, to solve; indeed, it is a problem that so far has largely eluded computer algorithms, and can thus be counted as an impressive feat of cognition.

Traditionally, the ability to separate multiple sound sources has been studied in two distinct ways. A great deal of basic research has been done with relatively simple stimuli that can be carefully manipulated in a controlled manner. For example, one of the earliest and most productive paradigms involves presenting two alternating tones whose features can be manipulated systematically in order to understand the contribution of different cues to the binding or segregation of the elements into a particular perceptual outcome (Carlyon, 2004; Pressnitzer et al., 2011). Among the most important of these cues is the frequency separation between the elements of the stream, independently of whether there is spectral overlap or not (Vliegen and Oxenham, 1999; Roberts et al., 2002), as well as the duration of the elements (Bregman et al., 2000); their spatial separation can also be relevant (Middlebrooks and Onsan, 2012), as can timbral features (Bregman and Pinker, 1978), and Gestalt principles of grouping (Bregman, 2015). The role of bottom-up cues such as these can be contrasted with schematic, or top–down cues, such as knowledge of the pattern to be segregated (Bey and McAdams, 2002; Agus et al., 2010; McDermott et al., 2011), and by attention (Thompson et al., 2011). These experimental approaches have offered a great deal of insight about variables that contribute to segregation, but because of the relative simplicity of the stimuli, they may not necessarily provide good generalization concerning how these many cues may be integrated into a real-world situation in which both the target stimulus and the competing background sources are highly complex, overlapping in time, and dynamically varying.

A complementary approach to understanding sound-source segregation derives from the need to characterize individual differences in the ability to segregate complex sound sources, in various populations. A number of tests based on the ability to hear speech sounds mixed with background noise at different signal-to-noise ratios (SNRs) have therefore been developed. For English-speakers, some of the most common clinical speech-in-noise (SIN) tests are the hearing-in-noise task (HINT), the QuickSIN, and the words-in-noise task (WIN), and for young children, the Pediatric Speech Intelligibility (PSI) test. The HINT comprises simple sentences such as ‘A boy fell from the window,’ which are presented in a background of speech-shaped noise (Nilsson, 1994). The QuickSIN uses somewhat more complex sentences like ‘A cruise in warm waters in a sleek yacht is fun’ embedded in multi-talker babble (Killion et al., 2004). In the WIN, single words are embedded in multi-talker babble (Wilson, 2003; Wilson et al., 2007). The PSI uses words and sentences appropriate for 3–6 years olds, which are presented with similar competing speech; the child indicates pictures that correspond to the perceived information (Jerger, 1987). These tests and adapted versions for populations with other linguistic requirements [e.g., Mandarin adult speakers (Wong et al., 2007); school-aged children (Reetzke et al., 2016); Mandarin-speaking young children (Zheng et al., 2009)] have proven to be enormously valuable, and have seen widespread use in the literature in different contexts, for example in studies of aging and hearing loss (Anderson et al., 2013; Shinn-Cunningham et al., 2013; Alain et al., 2014), in educational and developmental studies (Hornickel et al., 2011; Strait et al., 2012; Thompson et al., 2017), in relation to linguistic factors such as bilingualism (Reetzke et al., 2016), and in relation to musical expertise (Parbery-Clark et al., 2009). However, these tests largely lack certain useful features that would enable researchers to probe the contribution of different cues to segregation. That is, if one observes differential performance by an individual, or by a group, it is often difficult to know whether performance is affected because of enhanced or disrupted abilities to use one or another cue in a given situation.

As a concrete instance of this situation, consider the issue of speech-in-noise enhancements associated with musical training, which is often correlated with better performance on a variety of auditory tasks. Although there is now a growing consensus that such advantages do exist (Coffey et al., 2017b), and are associated with differences in auditory working memory (AWM) (Kraus et al., 2012), selective attention (Strait and Kraus, 2011), and measures of cognitive control (Amer et al., 2013), it is still unclear what drives the phenomenon, or why it is not always observed in a given study (Boebinger et al., 2015; Swaminathan et al., 2015). Part of the reason for this lack of understanding is that most commonly used and standardized SIN test instruments provide only an overall score, or a SNR or threshold at which a particular level of performance is achieved. These aggregate scores can be usefully compared between groups and to norms but do not offer insight into the separate components that might be affected, or not, by training or other interventions. Similar arguments apply to clinical populations, where a global deficit may be identified, but whose impact on different sub-skills may nevertheless not be clear.

Our goal in the present study was to develop a tool that would incorporate some of the valuable aspects of both of these approaches, while also filling what we perceive as a gap in the literature. Whereas the experimental studies allow evaluation of how different cues may contribute to sound-source segregation, they are typically not well-suited for application in clinical or other real-world settings, nor are they intended to offer much in the way of ecological validity. Conversely, the speech-in-noise tests focus exclusively on speech processing, which is clearly an important function, but they leave out aspects of stream segregation that may manifest in non-speech domains such as music; also, by focusing solely on speech, such tasks necessarily load on linguistic abilities and knowledge, which may be desirable in some contexts but not in others. In particular, if knowledge of the target language is affected by additional factors such as native vs. second language (Tabri et al., 2010), socioeconomic status (Slater et al., 2015), hearing deficits during development (Lieu, 2013), and language delays (Cunningham et al., 2001), then assessment of auditory segregation capacities will be more complicated.

Here, we present the Music-in-Noise Test (MINT) which was designed to offer a complementary way to measure auditory segregation ability, and to provide a means of investigating population and individual differences in the sub-skills that contribute to global stream segregation or HIN perception, such as spatial cues, visual cues, predictive cues, etc. Music has been noted as being an excellent platform to study complex stream segregation and integration processes (Pressnitzer et al., 2011; Disbergen et al., 2018) because, with the exception of monophonic musical genres, it exploits the various cues described above to achieve a variety of artistic effects via the combination of different lines of sound.

The MINT comprises five conditions that could be easily extended to explore additional auditory and multimodal cues, and top–down influences. The MINT uses a match-mismatch trial design in which a short instrumental melodic target is embedded in “multi-music” noise, to provide informational masking akin to the multispeaker babble often used in speech-in-noise tasks such as QuickSIN (Killion et al., 2004) and WIN (Wilson, 2003). The target+noise mixture is compared to a second melody, presented in silence, which can be either identical or different from the target, and the listener must indicate if it matched or not. The conditions that were implemented include (1) a baseline condition where the target+background mixture is presented first followed by the comparison melody in silence, with no additional cues (Baseline); (2) same as baseline but using a rhythmic pattern with no pitch variation as the target (Rhythm); (3) same as baseline but introducing a spatial cue to the target embedded within the mixture (Spatial); and (4) same as baseline but introducing a visual cue (pattern of lines that follow the melody) to aid detection of the target within the mixture (Visual). In a fifth condition, subjects hear the target melody first, the memory of which helps to predict incoming information in the mixture, which is presented second (Prediction). All conditions were tested at four different signal-to-noise levels (0, -3, -6, and -9 dB).

We report MINT data from 70 young adults with normal hearing who differed in their musical training, in order to demonstrate the task’s sensitivity to different aspects of HIN perception, its reliability, its relationships to measures of lower-level (e.g., pitch perception) and higher-level cognition (e.g., auditory working memory), and its relationship to a classical measure of speech-in-noise perception (HINT). Based on the literature reviewed above, we expected that the presence of additional cues (spatial, visual, and predictive) would enhance performance compared to the baseline condition; we also expected musical training to lead to better performance on all the conditions, with perhaps a stronger improvement on the visual task given its similarity to reading of musical notation. We assumed that the MINT task would correlate to some extent with HINT scores, given that some of the cognitive aspects of stream segregation would be in common across the two tasks, but we also expected there to be some unique variance measured with MINT. We explored how performance varied as a function of SNR, which we selected with the intention of generating a wide range of performance to maximize the sensitivity of the test. We also tested the hypothesis that AWM performance would correlate with the Prediction condition to a greater degree than with the Baseline condition, as the benefit of that condition is maximized by accurately holding in mind the target melody that is first presented in silence. Finally, we discuss possible applications of the task and its potential to facilitate comparability between designs and across populations, suggestions for using and extending the MINT (the materials for which are made freely available to the community), and questions raised by our exploratory results of musical and language experience presented here.

## Materials and Methods

### MINT Task Description

Subjects were asked to judge whether two target patterns presented sequentially were identical or not; on each trial, one target pattern was embedded in masking sound (see below) and the other was presented in silence. Each condition included 20 trials presented as a block (block order was randomized across subjects). Before the main experiment, four familiarization trials of each condition (i.e., Baseline, Rhythm, Spatial, Visual, Prediction) with the least difficult signal-to-noise levels were presented to the participant, preceded by written and spoken instructions (“We will play you two short musical excerpts. One of them will be played in background noise. Your task is to determine if the two melodies match or mismatch.”). Feedback was given for the familiarization task. The experimenter confirmed understanding of the instructions, and offered to answer questions before the graded task began.

Note that a full 20-item additional control condition in which both excerpts are presented in silence has been added to the updated version of the task, in order to exclude participants who are not capable of processing the musical content of the tasks (Peretz et al., 2002; Liu et al., 2015). These materials are now available and included in the online materials, but are not included in the data reported herein.

#### Stimuli

Short (3–4 s) musical excerpts were created in MIDI format and rendered in 44.1 kHz WAV format (‘electric grand piano’ timbre, MuseScore^1^). Stimulus tempo was 120 beats per minute and included 4–7 notes of varying duration. Stimulus length was selected in order to be similar to well-studied auditory match-mismatch task designs (e.g., Foster and Zatorre, 2010a), and not to overly tax working memory. For half of the stimuli, scrambled versions were created that used notes of the same pitches and the same distribution of note durations, but in a pseudo-randomized order (beginning and ending on pitches at the extremes of the frequencies used in the study was avoided); these scrambled versions constituted the mismatch foils.

#### Masking Sound

**FIGURE 1:**
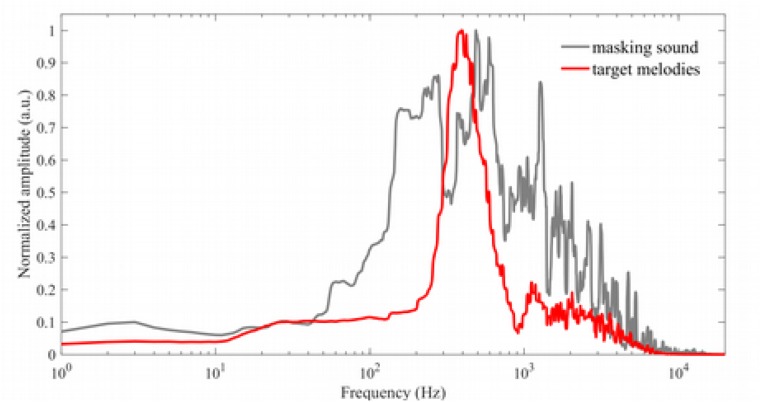
Spectra of the target (red) and multi-music masking sound (gray), demonstrating that the multi-music masking sound has broad spectral content, extending above and below that of the target stimuli (target spectrum shown here is averaged across all Baseline condition melodies).

Custom MATLAB scripts were used to modify and combine the stimuli (The Mathworks^2^). We downloaded MIDI files from a public repository of classical music scores^3^, altered their timbres in MuseScore2 in order to provide a range of timbres (amplitude normalized between -1 and 1). Four overlapping sound streams of polyphonic instrumental music were combined using Audacity to form 46 s of multi-music noise with a broad spectral content that extended above and below those of the stimuli; both informational and energetic masking is thus present (see Figure 1). A 47 s clip was taken in which all four pieces were represented, and which sounded relatively uniform. Excerpts (1 s longer than the melody) were randomly extracted and their ends tapered with raised 10 ms cosine ramps, to then be combined with the target stimuli, which were embedded in the noise starting 0.5 s from noise onset. The level of the multi-music masking sound was held constant, while the amplitude of the target stimuli was decreased by SNRs of 0, -3, -6, -9 dB. These values were determined by pilot testing to estimate a range of SNRs that would adequately capture the skill level of young healthy participants, while avoiding ceiling effects and limiting subject frustration. Four versions of each stimulus condition were prepared by combining the target with four different background stimuli at each of the four SNR levels, in order to create four separate versions of the task with randomized SNR-stimulus relationships. These four task versions were produced in advance to reduce online calculations that might introduce timing delays in some systems, and to allow the task to be used online. The interval between the melody in silence and melody in noise was 0.5 s. Musical excerpts embedded in noise were paired with matching excerpts in silence, or with their scrambled versions, to create an equal number of matching and mismatching stimulus pairs. Participants were allowed unlimited time to respond via a key press.

#### Conditions

**FIGURE 2:**
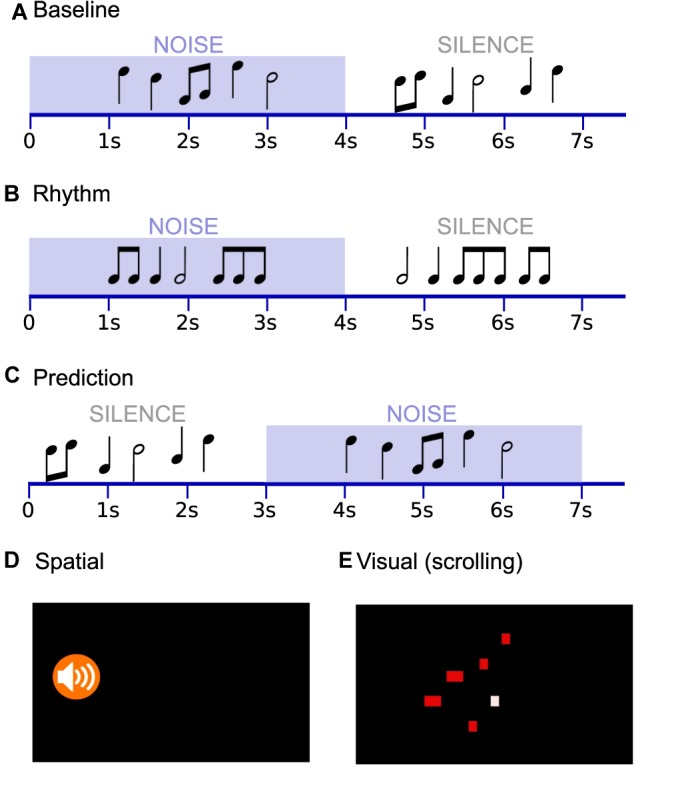
Music-In-Noise Task (MINT) conditions included five conditions: **(A)** Baseline, **(B)** Rhythm, **(C)** Prediction, **(D)** Spatial, and **(E)** Visual. In the Spatial condition, the time course resembled that of **(A)**, and an icon directed the listener’s attention to the side to which they should attend, before sound onset. In the Visual condition, the time course resembled that of **(A)**, and a scrolling graphic representation provided timing and approximate pitch cues (an example frame is shown). The given examples schematically represent mismatch trials.

Melodies and noise were combined to produce five conditions (i.e., Baseline, Rhythm, Prediction, Spatial, Visual), illustrated in Figure 2. In the Baseline condition, stimuli and noise were presented binaurally, first combined with noise and then in silence, with no visual cues. The Rhythm condition was similar except that the tones were all of identical pitch within the trial; only rhythmic variation distinguished matching and mismatching pairs. The Prediction condition was similar to Baseline, but the order of the stimuli was reversed such that the target melodies were first presented in silence, followed by the noise-masked pair. The Spatial and Visual conditions involved an audiovisual component. In the Spatial condition, the relative sound level of the melody and noise was adjusted in each ear to achieve the impression that the sound was coming from the left or right side (i.e., the melody was increased in one ear by +3 dB and the noise was decreased in the same ear by -3 dB such that the interaural level difference was 6 dB; the manipulation was inverted for the opposite ear). An icon at the beginning of the trial appearing on the left or right side of the screen directed the listener’s attention to the ear in which the melody was to be louder (Figure 2D); it then faded to a black screen. The target melody presented in silence was presented binaurally with equal loudness in both ears, as in the other conditions. For the Visual condition, a graphic representation of scrolling tones was created using a Python wrapper of freely available software^4^, see Figure 2E. A silent version of the video was recombined with the sound files using open source video editing software (Kdenlive^5^), such that the sound quality matched those of the other conditions. The visual representation was presented only during the sound-in-noise portion of the trial (so as to provide the visual cue during to help disambiguate target from background, but avoid task completion based exclusively on the visual representation).

### Participants

We recruited 70 healthy young adults (40 females, 30 males) with varying musical experience and training. The average age was 24.8 years (*SD* = 5.2, range: 18–46). The entire group was included for the research questions that examined musicianship as a continuum, and the relationships between behavioral measures, except where otherwise noted. To perform group analyses on the effects of musical training, we identified subjects who were currently practicing musicians (operationally defined as > 10 years of musical activity, and >4000 h of cumulative lifetime practice; *N* = 26; mean age: 26.2, *SD* = 5.1), and those who had fewer than 2 years of instrumental or vocal training and were not currently practicing were defined as non-musicians (*N* = 29, mean age: 23.9, *SD* = 5.4). One musician was no longer practicing, but was included in the musicians group due to extensive previous activity (>5200 h of practice). For these group analyses we therefore excluded individuals who fell in the intermediate range of these variables. The musician group had a heterogenous mixture of musical experience (total practice hours: mean = 9300, *SD* = 5800), with a variety of instruments (12 Keyboard, 5 String, 4 Woodwind, 4 Voice, 2 Percussion, 1 Brass); their mean age of formal training onset was 6.35 years (range: 3–13 years).

To perform group analyses involving the HINT, only subjects who reported being native English speakers were retained (*N* = 46; of these, 15 also were classified as musicians, and 17 were classified as non-musicians, excluding from group analyses 14 who fit in neither category). Conversely, to consider the effects of experience with multiple languages on the MINT, we also identified via self-report monolingual English-speakers (*N* = 15) and multilinguals (*N* = 55); in addition to English, multilinguals spoke a variety of languages (some people spoke more than one language): French (30), Spanish (4), Mandarin (4), Cantonese (2), German (2), Italian (2), Russian (1), Arabic (1), Catalan (1), Azerbaijani (1), Indonesian (1), and Kiswahili (1); in addition 24 individuals spoke English as a second language.

All subjects had normal or corrected-to-normal vision, and reported having had no history of neurological disorders. Pure-tones thresholds from 250 to 8000 Hz were measured to confirm auditory function (<25 dB SL pure tone hearing thresholds between 125 and 8000 Hz; Maico MA 728 audiometer; Minneapolis, MN, United States). Two subjects with slightly elevated binaural hearing thresholds (30 dB) were excluded from MINT and HINT analyses as these tasks may depend on the deficient frequency range. One subject was excluded from the MINT analysis for technical reasons. Subjects provided written informed consent and were compensated for their time, and all experimental procedures were approved by the Montreal Neurological Institute Research Ethics Board.

### Testing Session

Prior to the testing session, subjects completed the Montreal Music History Questionnaire to confirm eligibility and provide self-reported information about their musical and language experience (Coffey et al., 2011). Total cumulative practice hours were calculated by summation of practice and training hours reported for instrumental and vocal experience. Testing was conducted in a sound-attenuated audiometric booth and lasted ∼90 min. It began with an audiogram to verify basic auditory function, followed by the four behavioral tasks: a fine pitch discrimination task (FPD; Levitt, 1971), the MINT, a speech-in-noise task (HINT; Nilsson, 1994), and an AWM task (Albouy et al., 2017); see below for descriptions. Due to a technical problem, several participants had to complete the MINT on a different day. Participants were allowed to take breaks at any moment during the testing. A comfortable sound level was determined during pilot testing and kept constant for all subjects and tasks, which were all presented diotically (i.e., identical speech and masker in each ear) via headphones (ATH M50x Professional Monitor Headphones, Audio-Technica). For the MINT task, this corresponded to SPL that fluctuated between 70 and 80 dB (L_AF_) as measured with a Bruel & Kjær Model 2250 audiometer during the presentation of the melodic stimuli in silence.

### Behavioral Tasks

We measured speech-in-noise ability using a custom computerized implementation of the HINT (Nilsson, 1994) that allowed us to obtain a relative measure of SIN ability without specialized equipment. In the standard HINT, speech-spectrum noise is presented at a fixed level and sentence amplitude is varied with a staircase procedure to obtain a (single-value) SIN perceptual threshold. Our modified HINT task used a subset of the same sentence lists (Bench et al., 1979) and speech-spectrum noise, but presented 60 sentences, 10 at each of six difficulty levels (-2 to -7 dB SNR in steps of 1 dB SNR), an empirically determined range that is sufficient to capture HINT variability within healthy young adult populations in a single, averaged score (Coffey et al., 2017a). The sentences and noise were combined using custom scripts in MATLAB (The Mathworks). Stimuli were divided into blocks of five and block order was randomized by subject, such that each subject was presented with 12 blocks. Subjects were instructed to face away from the experimenter to avoid inadvertent feedback, and to repeat back what they had heard; proportion of correct words per sentence was calculated. No verbal or visual feedback was given. A single overall accuracy score as the proportion of sentences correctly repeated back to experimenter was calculated by averaging the accuracy across all SNR levels.

We measured FPD using a two-interval forced-choice task and a two-down one-up rule to estimate the threshold at 70.7% correct point on the psychometric curve (Levitt, 1971). On each trial, two 250 ms sine tones were presented, separated by 600 ms of silence. In randomized order, one of the two tones was a 500 Hz reference pitch, and the other was higher by a percentage that started at 7 and was reduced by 1.25% after two correct responses or increased by 1.25 after an incorrect response. The task stopped after 15 reversals, and the geometric mean of the last eight trials was recorded. The task was repeated five times, and the scores were averaged.

We used an AWM task that requires subjects to hold three sequentially-presented 250 ms tones (duration of 750 ms) in mind and reverse their order during a 2 s retention interval, and then judge whether a subsequently presented probe sequence had been correctly reversed, as reported as the ‘Manipulation Task’ in Albouy et al. (2017). Presentation software (Neurobehavioral Systems, Albany, CA, United States) was used to deliver stimuli and to register button presses. 108 trials were presented, from which an average accuracy score was derived.

### Analyses Concerning MINT Design

#### Information Content of Stimuli

To ensure that MINT subtasks did not differ in the average difficulty level of the target melodies used in each stimulus set, we used the software IdyOM (Pearce, 2005), which yields a measure of information content (IC) based on the degree of predictability within each stimulus. Higher values represent more predictable melodies (Pearce and Wiggins, 2012). We conducted a one-way ANOVA on the stimulus’ IC over the four conditions that included both timing and frequency information. The Rhythm condition was excluded from this direct comparison, as only the timing of notes varied between Rhythm stimuli (resulting in an overall lower IC).

#### Distribution of Scores

To verify that the MINT average scores were normally distributed across the group (*N* = 67), we inspected the frequency distribution (histogram) and quantile–quantile (Q–Q) plots (Ghasemi and Zahediasl, 2012). We also evaluated normality using the Shapiro–Wilk tests.

#### Internal Consistency of the MINT

We used the Spearman–Brown coefficient (ρ[s]) in order to assesses the split-half reliability of the MINT, which has been argued to be the most appropriate reliability statistic for a two-item (i.e., correct, incorrect) scale (Eisinga et al., 2013). The Spearman–Brown coefficient was calculated by first randomly dividing the 20 items in each of the five MINT sub-tasks in two groups 1000 times, for each of which Pearson’s correlation coefficients were calculated between the average score of each group and then averaged to produce a single correlation coefficient per MINT sub-task, to which the Spearman–Brown correction was applied. To assess the reliability of the MINT average score, we first averaged mean accuracy across MINT conditions, and then applied the same procedure.

#### Signal-to-Noise Ratio

We performed a repeated-measures ANOVA with SNR level as the repeated measure to determine whether the SNR level settings were sufficient to create a range of difficulty levels. For this analysis we included only data from the Baseline condition, in order to evaluate the influence of SNR unconfounded by presence of any additional cues.

### Analyses Concerning MINT Whole-Group

#### MINT Subtask Scores

The premise of the MINT subtask design is that each of the subtasks offers different information to the subject, as compared with the baseline condition. We conducted a one-way ANOVA over subtask averages in order to test for statistical differences in accuracy. In order to test whether the availability of additional cues would lead to better scores, we also contrasted Baseline < Prediction, Spatial and Visual, and Baseline > Rhythm in planned *post hoc t*-tests (Bonferroni-corrected for multiple comparisons).

#### Relationships Between MINT and Supplementary Behavioral Measures

To test the hypothesis that the MINT reflects some of the skills common to speech-in-noise perception, we performed a Pearson’s correlation between MINT average scores and HINT scores on subjects who reported English to be their native language (*N* = 46). To evaluate the relationship between lower-level and higher-level auditory perception and manipulation skills, we calculated Spearman correlations between the MINT and fine-pitch discrimination ability and AWM, respectively. These analyses were not language-dependent and thus did not exclude non-native English speakers (*N* = 67). These analyses serve to validate the MINT task as a basis for exploring the roles of factors such as experience and expertise, including musicianship and language experience that are considered in the following sections.

### Analyses Concerning the Effects of Expertise

#### Effects of Musical Training

To test the effects of musical training, we first examined the entire group using a series of Spearman correlation (*r*[s]), to avoid bias by outliers (Bonferroni-corrected for multiple comparisons), entering hours of practice as the independent variable. In order to examine musical training effects as a function of SNR, we compared the highly trained musician group with the untrained group on the MINT, using the Baseline condition only so as to focus on the basic task, without any additional cues. We carried out a 2 (group) × 4 (SNR) ANOVA, including multilingualism as a covariate, so as not to confound musical training with language experience.

#### HINT vs. MINT by Group

To compare the effects of musical training on both the MINT and the commonly used speech task, the HINT, we entered scores of both tasks into an ANOVA, with musical training as the group variable and task as the repeated measure, including only English native speakers in this analysis.

#### MINT Subtasks With Musicianship

In order to test the effects of musical training as a function of the MINT subtasks, we ran a 2 (group) × 5 (conditions) repeated measures ANOVA, with multilingualism as a covariate. In order to evaluate the possible contribution of AWM, we then entered performance on our AWM task as an additional covariate to this ANOVA.

#### Effects of Multilingualism

In the previous analyses, bilingualism was included as a covariate, in order to ensure that musical training effects were not confounded by any group differences in linguistic experience. In order to explore possible effects of multiple language knowledge on MINT performance independently of musical training, we used only our sample of non-musicians, and compared MINT performance within this group, dividing them into mono- and multi-linguals.

## Results

### MINT Design Features

#### Information Content of Stimuli

The mean and standard deviation of IC for each condition were as follows: Baseline (mean = 7.26, *SD* = 1.02), Rhythm (mean = 6.00, *SD* = 1.01), Prediction (mean = 6.83, *SD* = 0.73), Spatial (mean = 6.68, *SD* = 0.48), Visual (mean = 6.63, *SD* = 0.77). There were no statistically significant differences between information content of stimuli between MINT subtasks as determined by one-way ANOVA (*F*[3,17] = 1.960, *p* = 0.158), suggesting that differences in accuracy scores between conditions cannot be accounted for by differences in predictability of the musical stimuli.

#### Distribution of Scores

The mean MINT average proportion correct across all subjects was 0.78 (*SD* = 0.09). Histogram, Q–Q plots were consistent with those expected of a normal distribution, and Sharpiro–Wilk’s tests were non-significant. The distribution of HINT average scores was not significantly different from the normal distribution, but AWM and FP scores were; for this reason we have used non-parametric statistics when these latter measures are concerned. Percentile scores based on the fitted normal distribution are provided in Table 1 to allow assessment of individual performance.

**Table 1:** Percentile scores based on the fitted normal distribution.

Percentile	MINT average (proportion correct)	Percentile	MINT average (proportion correct)
5	0.52	50	0.78
10	0.55	55	0.81
15	0.58	60	0.84
20	0.61	65	0.86
25	0.64	70	0.89
30	0.67	75	0.92
35	0.70	80	0.95
40	0.72	85	0.98
45	0.75	≥90	1


#### Internal Consistency

Spearman–Brown corrected correlation coefficients for the MINT subscales were as follows: Pitch: ρ[s] = 0.35, Rhythm: ρ[s] = 0.56, Prediction: ρ[s] = 0.40, Spatial: ρ[s] = 0.46, and Visual: ρ[s] = 0.48. For the MINT overall average, ρ[s] = 0.82. Split-half reliability scores indicated that the MINT average score had relatively high internal consistency. As expected, the subscales had lower split-half reliability values. Each question is intended to measure the same underlying construct (i.e., ability to perform sound-noise separation, given a set of cues), the varying difficulty levels between questions introduce response variability that sets an upper limit on the split-half reliability estimates for the smaller subtasks.

#### Signal-to-Noise Ratio

The accuracy level in the Baseline condition differed significantly across SNR level (*F*[3,63] = 26.52, *p* < 0.001), indicating that the signal-to-noise level settings were sufficient to create a range of difficulty levels. Performance for each condition followed an orderly pattern, decreasing as a function of decreasing SNR. The mean accuracy (proportion correct) was as follows: 0 dB SNR, 0.86 (*SD* = 0.19); -3 dB, 0.78 (*SD* = 0.17); -6 dB, 0.71 (*SD* = 0.25); -9 dB, 0.53 (*SD* = 0.22).

### MINT Whole-Group Outcome

#### MINT Subtask Scores

The mean accuracy scores for each condition were as follows: Baseline = 0.72 (*SD* = 0.12); Rhythm = 0.70 (*SD* = 0.14); Prediction = 0.83 (*SD* = 0.12); Spatial = 0.83 (*SD* = 0.10); and Visual = 0.82 (*SD* = 0.12). A one-way ANOVA revealed a significant main effect of subtask (*F*[4,63] = 29.27, *p* < 0.001; $\eta_{p}^{2}$ = 0.65). *Post hoc* tests revealed that additional cues increased performance significantly over Baseline, for the Prediction condition [*t*(66) = 6.85, *p* < 0.001], the Spatial condition [*t*(66) = 7.91, *p* = 0.001], and the Visual condition [*t*(66) = 5.65, *p* = 0.001]. The removal of pitch information in the Rhythm condition did not produce significantly lower scores as compared to the Baseline condition [*t*(66) = -1.68, *p* = 0.84]. These results indicate that subjects are able to make use of additional predictive, spatial, and visual cues when available, but that performance is not greatly affected by the absence of pitch variability.

### Relationships Between MINT and Supplementary Behavioral Measures

**FIGURE 3:**
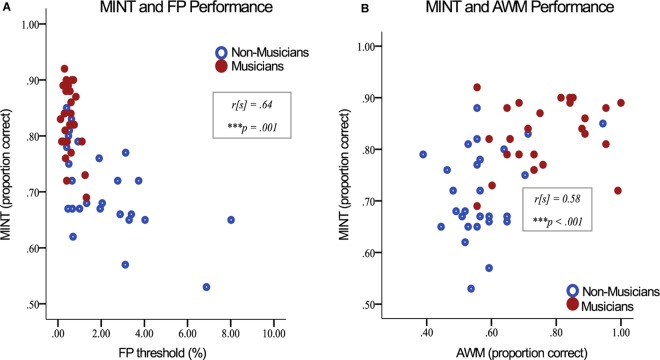
Music-In-Noise Task average score vs. **(A)** Fine pitch discrimination ability, and **(B)** auditory working memory (AWM) performance. Only musicians and non-musicians are included in this illustration in order to visually emphasize group differences.

The MINT average score was significantly correlated with AWM performance (*r*[s] = 0.52, *p* < 0.001; *N* = 67; when only members of the musician and non-musician groups are included, *r*[s] = 0.58, *p* < 0.001; *N* = 55), fine pitch discrimination (*r*[s] = -0.62, *p* < 0.001; *N* = 67; when only members of the musician and non-musician groups are included, *r*[s] = -0.64, *p* < 0.001; *N* = 55), and for English-speakers, MINT was significantly correlated with HINT scores (*r*[s] = 0.41, *p* = 0.006; *N* = 44; Figure 3). We looked more closely at the relationship between AWM performance and MINT, and observed that the strongest relationship was with the prediction condition (*r*[s] = 0.56, *p* < 0.001); there was no significant correlation with the baseline condition (*r*[s] = 0.18, *p* < 0.15). The difference between these two correlations was significant (*z* = 3.18, *p* < 0.001). The other conditions had significant correlations with AWM at a moderate level: rhythm (*r*[s] = 0.41, *p* < 0.001); spatial (*r*[s] = 0.29, *p* < 0.02); visual (*r*[s] = 0.49, *p* < 0.001).

### MINT Effects of Expertise

#### Effects of Musical Training

**FIGURE 4:**
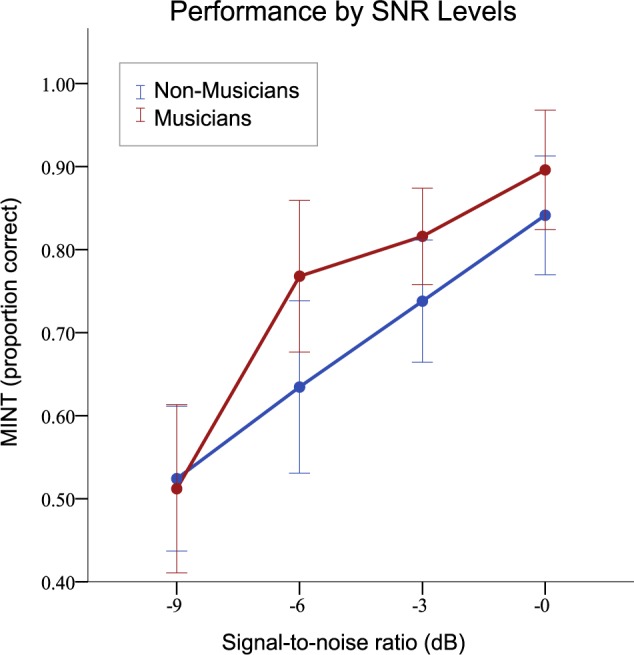
Musical training effects as a function of signal-to-noise ratio (SNR) of MINT stimuli, averaged across all MINT conditions. Musicians had significantly better scores overall. Error bars indicate 95% confidence interval.

We found significant correlations between cumulative hours of musical practice and the following measures: MINT scores *r*[s] = 0.531, *p* < 0.01 (two-tailed), fine pitch *r*[s] = -0.491, *p* < 0.01 (two-tailed), and AWM: *r*[s] = 0.611, *p* < 0.01 (two-tailed). In all cases, musical training was associated with enhanced performance. On a 2 (group) × 4 (SNR) ANOVA with multilingualism entered as a covariate, we observed a large main effect of SNR (*F*[3,50] = 21.47, $\eta_{p}^{2}$ = 0.59, *p* < 0.001), and also not surprisingly, the musicians performed significantly better (*F*[1,52] = 4.90, $\eta_{p}^{2}$ = 0.09, *p* < 0.03); but there was no interaction between these two variables, indicating that the musician advantage held across the different SNRs (Figure 4).

#### HINT vs. MINT by Group

**FIGURE 5:**
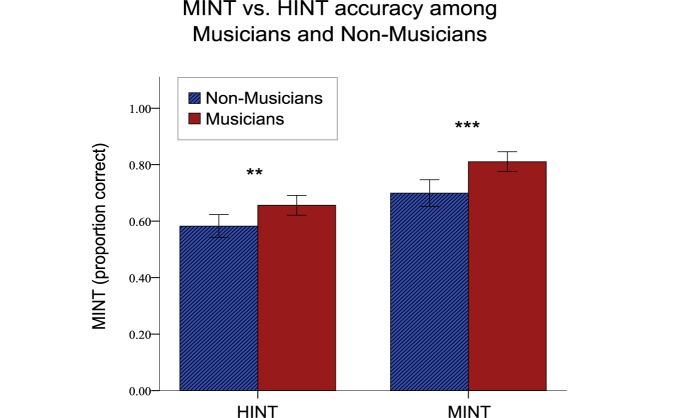
Musical training effects on MINT and HINT scores. Musicians demonstrated a perceptual advantage when both linguistic and musical stimuli are presented in noisy conditions (^∗∗∗^*p* < 0.001, ^∗∗^*p* < 0.01). Error bars indicate 95% confidence interval.

Our analysis of HINT and MINT scores on English native speakers revealed a significant main effect of Musicianship (*F*[1,29] = 23.06, *p* < 0.001), $\eta_{p}^{2}$ = 0.44, representing a mean gain of 9.5% (p < 0.001). There was also a significant Task effect: *F*[1,29] = 54.87 (*p* < 0.001) $\eta_{p}^{2}$ = 0.65 as the HINT is a slightly more difficult test overall. Importantly there was no Musicianship by Task interaction (*F*[1,29] = 1.466, *p* = 0.236, $\eta_{p}^{2}$ = 0.05) indicating that musicians outperformed non-musicians to an equal degree on both HINT and MINT (see Figure 5).

#### MINT Subtasks With Musicianship

**FIGURE 6:**
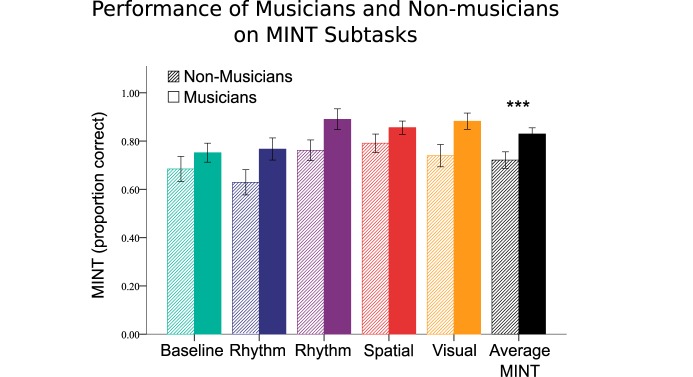
Musical training effects on MINT scores by subtask and grand average. Error bars indicate 95% confidence interval.

In order to test the effects of musical training as a function of the MINT subtasks, we ran a 2 (group) × 5 (conditions) repeated measures ANOVA, with multilingualism as a covariate (Figure 6). In addition to the expected main effects of musical training (*F*[1,52] = 33.58, *p* < 0.001), and MINT subtasks (*F*[4,49] = 23.59, *p* < 0.001), there was also an interaction between the two variables (*F*[4,208] = 2.83, *p* = 0.026). *Post hoc* tests indicated that performance was enhanced for the Prediction (*t*[54] = 6.01, *p* < 0.001), Spatial (*t*[54] = 6.56, *p* < 0.001), and Visual (*t*[54] = 5.39, *p* < 0.001) subtasks compared to the Baseline condition. In order to evaluate the possible contribution of AWM, we entered performance on our AWM task as an additional covariate to this ANOVA; when we did so, the main effects remained, but the interaction effect became non-significant (*F*[4,200] = 1.44, *p* = 0.224).

#### Effects of Multilingualism

**FIGURE 7:**
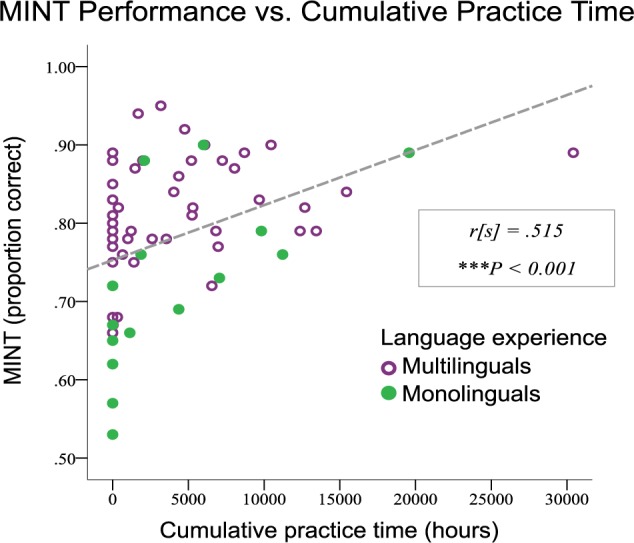
Music-In-Noise Task performance vs. cumulative practice hours, wherein linguistic experience is indicated by color and symbol. Irrespective of musical training, multilinguals had superior performance on the MINT.

Multilinguals significantly outscored the monolinguals in the non-musician group in a direct comparison (*t*[27] = 3.41, *p* < 0.002). This pattern is illustrated in Figure 7, which shows scores for mono- and multi-linguals as a function of total hours of musical practice; the subjects included in the analysis would be the ones of the left side of this scatterplot (i.e., zero hours of cumulative musical practice time). We also observed an effect of multilingualism in the entire group in the 2 × 5 repeated measures ANOVA reported above in the section entitled ‘MINT Subtasks with musicianship’ (*F*[1,52] = 13.30, *p* = 0.001), in which musicianship was also included. We additionally tested whether there was an interaction between linguistic group and MINT subtask in a 2 × 5 repeated measures ANOVA wherein linguistic experience was the between-subjects variable and cumulative practice hours was included as a co-variate. The interaction was not significant (*F*[4,61] = 1.674, *p* = 0.168), suggesting that the benefit of multiple languages is not specific to subtask(s).

## Discussion

### Validation and Design Features

The goal of this study was to develop and present behavioral findings from the MINT, an instrument to measure individual differences in perceptual abilities to segregate target musical sounds from background tonal patterned noise, and which also includes a means to evaluate the contribution of different types of information (visual, spatial, or predictive) to that process. Our analyses of the MINT’s psychometric properties support its reliability, validity, sensitivity, and suitability for testing contributors to perceptual segregation ability. We also found that scores were approximately normally distributed, allowing for parametric statistical analyses to be applied. We here provide normative data for one specific implementation of the MINT in healthy young volunteers, and include percentile scores for assessment of individual performance (Table 1). But the test is conceived as modular, such that it could be modified by different investigators to meet their needs. It should also be straightforward to apply to other groups, including clinical populations, with modifications as necessary to the difficulty level (i.e., SNR). An additional feature that is included in the available on-line materials, but did not form part of the present study, is a condition in which the target is presented twice, without any noise masking. This simple melody discrimination task can be used as a screening procedure, in the case where one wants to test a clinical population suffering from general cognitive impairment, or amusia, or any group where it may be necessary to determine if the basic discrimination task can be performed sufficiently well in the first place, in order to then assess HIN ability.

Music-In-Noise Task scores were internally consistent. The split-half reliability of the whole test was ρ = 0.82. The reliability of each of the five conditions taken separately is lower, as expected due the fewer numbers of trials involved; they should therefore be used with some caution to infer impairments in any given individual for instance. However, the test could easily be adapted to include more items for any given condition if there is an experimental question that requires further data from one particular condition. The target stimuli were carefully balanced across conditions such that their information content, as determined by the IdyOM model (Pearce, 2005), did not differ significantly between the stimuli used across the four conditions, thus ensuring that performance differences across conditions were not due to difficulty level of the target melodies. Another desirable design feature is that the MINT spans a large overall range of performance, making it sensitive to individual differences. An additional advantage of the MINT over tasks such as HINT that require evaluation of a verbal response, is that the scoring is completely objective and straightforward, requiring no judgment of correctness on the experimenter’s part. It would also be possible to obtain measures of latency from the MINT if that were desirable.

When MINT results were inspected across the four SNRs used, accuracy scores differed significantly across the levels in an orderly manner, and no ceiling effect was observed in the easiest condition, suggesting that the difficulty manipulation was successful and the range of difficulty levels used here captured the range of abilities in our population, making it sensitive to variation (such as from musical training), or presumably also from disorders. However, even though our test population consisted of healthy normal-hearing young adults, the average score at the most difficult SNR was close to chance levels (proportion correct of 0.53), suggesting that for groups with impaired hearing or other impairments, it may be appropriate to omit the most difficult SNR (-9 dB) and/or to add easier SNR conditions, which should be straightforward to do as we provide the separate target and noise sound files, allowing for modification as required. We here opted to use informational masking (Kidd et al., 2008) via a complex, changing background stimulus (which we term “multi-music noise”) for two reasons. First, it is most comparable to the multi-speaker babble used in some measures such as the QuickSIN. Second, it is thought that informational masking makes greater demands on higher-order cognitive processes that we were most interested in, as compared to energetic maskers (typically white or pink noise), which are more sensitive to peripheral hearing mechanisms. Informational masking has also been shown to demonstrate effects associated with musical training (Oxenham et al., 2003). We here provide both the target stimuli and maskers separately, allowing investigators to manipulate each of them as they wish.

One of the principal design features of the MINT is its ability to evaluate the contribution of different sources of information. We found that the addition of Spatial and Visual cues enhanced performance, as expected from the literature in speech and noise (Grant and Seitz, 2000; Ross et al., 2006). The Rhythm condition was equally difficult to the Baseline condition, indicating that rhythm cues on their own are sufficient to perform the task (but see below for effect of musical training on this task). The advantage of the MINT, as compared to other available tests, is that the contributions of different cues may be assessed quantitatively using the same materials, allowing for direct comparison of the influence of these variables. We also implemented the Prediction condition, where the target is presented in the reverse order to the other conditions, that is, prior to the embedded sound. Again, as expected from the literature (Bey and McAdams, 2002) hearing the target first raises performance levels significantly. This particular subtask should be especially valuable to evaluate top–down mechanisms of disambiguation as well as working memory function; indeed, the score on the Prediction task was the most highly correlated with the score on the AWM task, indicating that working memory is particularly important for this condition, unlike the Baseline condition where there was no significant correlation with working memory. The AWM task also correlated significantly, albeit at a lower magnitude with the other conditions, indicating that working memory capacity plays a role when integrating additional cues into task performance. It should also be noted that the thresholds of fine pitch discrimination also correlated with average MINT performance. This relationship underscores that both low-level abilities, such as pitch discrimination, as well as higher-order skills, such as capacity to manipulate information in working memory, play a role in stream segregation, as has been pointed out repeatedly in the literature (e.g., Anderson et al., 2013).

### Relationship to Linguistic Variables

We also evaluated the MINT’s relation to a commonly used speech-in-noise task (HINT), taking only native English speakers so as to not underestimate HINT performance in non-native speakers. MINT and HINT scores significantly correlated, as predicted on the basis that there are some shared mechanisms between the two tasks. This finding serves as partial validation of the MINT, based on the known relevance of HINT for many applications. However, HINT scores only explained a modest proportion of MINT scores (17%), which means that MINT is sensitive to other sources of variance not captured by HINT. This differential sensitivity may be related to the difference in available cues known to influence auditory stream segregation in general, in particular, spatial, visual, and predictive cues, which are not separately evaluated in the HINT [although there is a validated option to present the HINT with spatial information (see Nilsson, 1994), the more common binaural version was used here]. As well, since MINT is non-verbal by design, it is likely that a good part of the difference between the two tasks relates to the presence or absence of linguistic information, which may influence different individuals differently. Indeed, the non-verbal nature of MINT may be an advantage in testing certain populations precisely because it does not depend on knowledge of any one language, thus allowing it to be used in a wider sample of individuals than is possible with speech-in-noise tasks which of course require knowledge of the target language. However, we do not claim that the MINT is in any way culture-free, because the materials used are derived from Western music, and hence exposure and implicit knowledge of Western music would be expected to affect the results. These analyses do nonetheless demonstrate the sensitivity of MINT to factors known to affect hearing in noise ability in a range of studies, and support the use of the MINT as a complement to other existing tests for exploring the roles of factors such as experience and disorders.

To test the possible influence of multilingualism, we broke down the sample into mono- and multi-lingual speakers, taking only those classed as non-musicians, since there were insufficient numbers of individuals to examine the interaction of musical and linguistic expertise. Despite the limited sample size in this subgroup (*N* = 29), we observed a highly significant effect such that multilinguals outperformed monolinguals on the overall MINT score. Similarly to musicians, bilinguals and multilinguals are exposed to a rich and varied repertoire of auditory inputs integrated with other modalities (Mechelli et al., 2004). Most relevantly, Krizman et al. (2012) found that bilinguals showed an enhanced physiological encoding of the fundamental frequency of sounds compared to monolinguals, similar to the enhanced encoding associated with musical training; thus, this mechanism could explain the effect we observed since it would lead to better encoding of pitch and pitch relationships, making it easier to detect a target melody embedded in noise. However, this bottom-up enhancement may not be the only reason for such an advantage, since learning multiple languages has many effects on cognition, including some reported enhancements to executive function (for review see, Costa and Sebastián-Gallés, 2014), which could have influenced performance on our task via top–down mechanisms. The present study was not designed specifically to test for the effects of bilingualism or multilingualism, which would require a more rigorous determination of language proficiency, age of start, current use, and other demographic and linguistic factors. But our finding of multilingual enhancement on a non-verbal task is of interest because the majority of research on bilingualism has focused on advantages for linguistic tasls; possible transfer effects from language to music or non-verbal processing may be an interesting area for future study, and the present findings suggest that the MINT could be used to explore these questions in a more detailed manner.

### Relationship to Musical Training

As predicted, musical training was associated with enhanced overall MINT performance; this effect was evident when comparing two groups that differed maximally in their musicianship, excluding intermediate cases (Figure 5), as well as when musical training was treated as a continuous variable, based on cumulative hours of training (Figure 7). The musical training effect was constant across SNR levels, since it did not interact with that variable. Thus, it is a fairly robust phenomenon, and one that is consistent with several prior studies that have studied stream segregation in non-verbal contexts (Zendel and Alain, 2009), although none of them specifically used real musical materials.

Musical training interacted with the different conditions (Figure 6): the group difference was greatest for the Rhythm, Prediction, and Visual conditions. The effect seen for the Rhythm condition is best interpreted not as an enhancement in musicians, since their scores were similar for the baseline condition and the rhythm condition (0.75 and 0.76 proportion correct, respectively), but rather as reflecting greater difficulty on the part of non-musicians, whose scores on the Rhythm test (0.64) were the lowest of any condition, and below even the Baseline condition (0.70). It is likely that the absence of pitch variation in this condition, constituted a more impoverished stimulus for the non-musicians, who were unable to use the temporal cues alone as effectively as the musicians could.

Conversely, the musician advantage for the Visual and Prediction conditions is best seen as a true benefit of musical training. It is not very surprising that the Visual condition would facilitate musicians’ ability to segregate the target from background, since the visual stimuli used were similar to musical notation, with which musicians would have a lot of experience. But our visual stimuli were also physically closely related to the target melody (not purely symbolic), as the onsets and durations mapped directly to the onsets and durations of the target tones, and furthermore there was a direct mapping of pitch to position on the screen; thus these cues were available to all listeners to disambiguate the target item using top–down cues, and indeed there was a global improvement across all participants, not just musicians. However, musical training led to a better ability to make use of such cues. There is already evidence that music notation can help musicians to segregate a melody from a background (Marozeau et al., 2010), consistent with the present data. Our finding is also consistent with broader findings favoring multimodal integration in musicians: several studies have shown enhanced behavioral and neurophysiological responses to synchronous audiovisual stimuli in musicians compared to non-musicians (Musacchia et al., 2007; Lee and Noppeney, 2011), which is likely the mechanism to explain at least part of the musical training advantage observed on these particular tasks.

Regarding the Prediction condition, it also resulted in a proportionally greater gain amongst musicians as compared to non-musicians. One explanation for this relative advantage is that the Prediction task requires good working memory ability in order to retain the target melody in mind while the noise mixture is presented. Evidence that this factor plays a role comes from the high correlation between our independent measure of AWM and performance on this task (*r*[s] = 0.56), which was the highest correlation obtained with this task across the five conditions. Moreover, musical training was associated with higher performance in the AWM task, as expected from prior studies (Foster and Zatorre, 2010b). Finally, when AWM performance was included as a covariate in the analysis, the interaction between musical training and task disappeared, suggesting that the principal reason that musicians benefit disproportionally in the prediction condition is due to their enhanced AWM ability.

We also evaluated how musical training affected scores on the MINT relative to scores on the HINT, which involved understanding sentences in noise (for this analysis only native English speakers were included so as not to confound linguistic knowledge with musical training). We were able to replicate in our sample the musician enhancement of performance for speech-in-noise that has often been reported in the literature [though not every study has observed it (see Coffey et al., 2017b)]. Indeed, the degree of enhancement associated with musical training was similar for the two tasks, suggesting that the stream segregation mechanisms available to musician enable better processing of both musical and speech cues. This conclusion is consistent with other findings that not only are musicians better at speech-in-noise, but also at segregating one speech sound from another (Başkent and Gaudrain, 2016; Puschmann et al., 2018), suggesting a general ability to extract acoustical cues that are relevant not only for musical sounds but also for speech tracking (e.g., amplitude envelope—see Puschmann et al., 2018). But as repeatedly noted in the literature, there may also be more cognitive components that contribute to the enhancements often seen in speech-in-noise, and the relative balance of these skills may change from one situation to another. For instance, Du and Zatorre noted that musician-related improvement in speech-in-noise was associated with better decoding accuracy in brain imaging data from auditory cortex in high SNR conditions, suggesting more perceptual segregation abilities, whereas under low SNR conditions better decoding accuracy was seen in frontal and motor brain regions suggesting top–down mechanisms (Du and Zatorre, 2017). Thus both mechanisms likely contribute depending on the availability of different cues and specifics of the musician sample being tested. One advantage of the MINT is that the presence or absence of particular kinds of cues can be manipulated via the Prediction, Spatial, and Visual conditions to evaluate the contribution of these factors more specifically. It would be of interest in future to develop a similar set of conditions for a speech-in-noise application.

## Conclusion

The MINT is an instrument to measure auditory stream segregation ability using relatively naturalistic non-linguistic materials, while at the same time providing good experimental control. Here we give an overview of its psychometric properties, provide some normative information, and demonstrate its sensitivity to individual variables such as musical training and language experience. We also show how the removal or addition of various cues, via the five different subtests, affects performance, allowing for the assessment of the contribution of these cues to different situations. We hope that the community will find it useful either in its current form or with modifications, both of which should be facilitated by the availability of all the materials that go into the test.

## Data Availability

The datasets, stimuli and scripts created for this study are available online and by request to the corresponding author^6^.

## Author Contributions

EC and RZ designed the task. EC created stimulus generation scripts, which were further modified by undergraduate student Ah Ryung Lim, who also collected pilot data during task development. EC, IA-B, and XZ collected data, along with undergraduate student Nicolette Mogilever. IA-B, XZ, and EC prepared the data for analysis. IA-B conducted the statistical analyses and with EC, prepared the figures. EC, RZ, and IA-B wrote the paper, with contributions from XZ.

## Conflict of Interest Statement

The authors declare that the research was conducted in the absence of any commercial or financial relationships that could be construed as a potential conflict of interest.

## Footnotes

1 www.musescore.org

2 www.mathworks.com

3 http://www.piano-midi.de/

4 www.musanim.com

5 www.kdenlive.org

6 www.coffeylab.ca/open-science
